# Cyclones and Sickness in India

**Published:** 1865-06

**Authors:** 


### CYCLONES AND SICKNESS IN INDIA.

Within the past few months, two cyclones of terrible severity have occurred in the bay of Bengal. In a brief notice of these most remarkable rotary storms, these gigantic whirlwinds, we shall not be departing far from the strictly professional range to which we limit ourselves in this journal; for these tornadoes are not only attended with great destruction of life, but are also the fruitful cause of disease and death. They generally occur about the time of the change of the monsoons, that is, in the months of October and April.

The first of these two cyclones occurred on the fifth of October last. Its central force seems to have been felt at the head of the bay in the region of Calcutta. The waters of the bay were piled up to a height of thirty feet, and driven with fury over the island of Saugor, and other low lands at the mouth of the Hoogly. It flooded the entire delta of the Ganges known as the Sauderbunds; it swept up the river to Calcutta and far beyond. All up the river, the population on both sides has literally been "carried away as with a flood." It was at first reported that 12,000 persons had perished by these inundations. This was regarded as an exaggeration, and disbelieved in England, but subsequent inquiry proved that this estimate was very far below the truth. It is now ascertained that more than five times that number perished by drowning or in other ways. In the island of Saugor alone 7,000 persons are known to have been swept away. An eye witness says:—"The Sauderbunds are swept bare of every living thing, and every habitation. The river and the land were strewed with dead bodies of men, cattle, and even snakes. The river was so full of dead bodies of all kinds, as to impede the progress of the steamer. A missionary speaks of a police officer, who in coming from Diamond Harbor, (near the mouth of the Hoogly,) to Calcutta, made his way with immense difficulty, and counted over 5,000 corpses on his route. Of the 200 ships and steamers at Calcutta, more than 100 were destroyed, and generally with all on board. Four hundred and ten houses, well built of masonry, were utterly ruined, and more than 1,600 seriously damaged. Of the small native houses in the city, 40,000 were destroyed. More than 1,000 persons perished by the falling of houses alone. A recent estimate places the loss of life by this cyclone at 60,000!

By a private letter we have notices of a second cyclone, the principal force of which seems to have been felt on the coast to the southward of Calcutta. At Marsulipatam, a large city on the western shore of the bay, 20,000 persons were either killed or drowned during the storm. Thirty-five out of sixty girls in a boarding school of the Church Missionary Society in that city perished.

As was anticipated, disease had already commenced its ravages in the form of cholera, small pox, and fever. The latter which often prevails as an epidemic in those low, marshy districts, is now depopulating whole districts. The poor natives die on all hands without hope of assistance, and without medicine. There appear to be no means to stop the progress of the diseases which are now devastating the country. The native feels ill, wraps himself in his blanket, says it is his *fate*, and so perishes.

All this is sad enough when reviewed at this distance, but it is not without its personal significance and interest to us; for India has always had the reputation of being the seed-plot of disease. The Asiatic cholera, one of the most fearful scourges of our race, claims India for its birth-place.—*March Editorial Med. and Surg. Reporter.*

